# Supplementary material for: Navigating pathways to develop self-compassion in sport, dance, and music: Qualitative insights from volunteer participants on balancing criticism and compassion, the pivotal influence of the social environment and strategies to overcome setbacks
Source: PLoS One. 2025 Jun 25;20(6):e0326612. doi: 10.1371/journal.pone.0326612 (PMC12194077; doi:10.1371/journal.pone.0326612)
Supplement: S1 Table — It is the interview guide used for the 27 semi-structured interviews. (DOCX) [file pone.0326612.s001.docx]

# **Supporting information**

**S1 Table. Interview guide.** It is the interview guide used for the 27 semi-structured interviews.

| **Initial instructions/opening question**: Tell me about your training sessions/classes with your coach/music or dance teacher and how you behave towards yourself during your training. | | |
| --- | --- | --- |
| **Themes** | **Subtopics** | **Follow-up questions** |
| **Training sessions and environment** | Social environment, conditions | - Who is usually present during your training sessions?  - How are things going with the others? |
|  | Personal characteristics during training | - What kind of athlete/musician/dancer are you with yourself?  - How do you talk to yourself when you're practising?  - Are there any periods and/or key moments in your practice when you are harder on yourself? |
|  | Emotions | - What kinds of emotions do you feel during your training ? |
| **Thematic question** | Can you tell me about a recent event/situation when you felt you were being rather hard on yourself? | |
| **Self-compassion** | Attitude towards oneself (self-respect) | - What feelings did you have at that time?  - What thoughts come to mind at times like those? |
|  | Level of demand, reaction to difficulties | - How do you overcome obstacles and the mistakes you make?  - What strategies do you use to overcome these obstacles? |
|  | Positioning in relation to others (common humanity) | - How do you position yourself in relation to others?  - Do you feel well supported or alone in the face of your difficulties? |
|  | Effects of one's own attitudes on one's practice and relationships | - At times like these, what do you need or miss?  - At times like these, what or who can you lean on? |
| **Phrases on self-compassion: comparing them to their own experience** | | |
| **Thematic question** | What roles do your coach/music or dance teacher and the wider community play in your day-to-day practice? | |
| **Relationships/interactions with those around them (coach, parents, peers)** | Peers, coach, parents | - How are your training sessions with your coach/music or dance teacher going?  - What role do your parents play?  - What kind of links do you have with other athletes/musicians/dancers? |
|  | Interaction with others | - How are decisions made about your practice?  - How does your coach/music or dance teacher react and act with you?  - How do you communicate with each other? |
|  | Assessing the attitudes of those around you | - What do you need from those around you?  - How would you like them to act in relation to your practice? |
| **Coaches' or teachers' testimonials**  What do these sentences mean to you? What do you think about when you read them? | | |
| **Closing question**: From whom and how would you like to get help in developing self-compassion skills: online, via your coach/music or dance teacher, personal guidance, tips...? | | |
