# Supplementary material for: Navigating pathways to develop self-compassion in sport, dance, and music: Qualitative insights from volunteer participants on balancing criticism and compassion, the pivotal influence of the social environment and strategies to overcome setbacks
Source: PLoS One. 2025 Jun 25;20(6):e0326612. doi: 10.1371/journal.pone.0326612 (PMC12194077; doi:10.1371/journal.pone.0326612)
Supplement: S2 Table — These are the three sentences presented to the participants during the interviews. (DOCX) [file pone.0326612.s002.docx]

# **Supporting information**

**S2 Table. Sentences representing the three components of self-compassion.** These are the three sentences presented to the participants during the interviews.

| **Self-kindness** | "If I think back to the situation in which I was hard on myself, I am aware of my thoughts and emotions and avoid over-identifying with them." |
| --- | --- |
| **Common humanity** | "If I think back to the situation in which I was hard on myself, I behaved in the same way I would have with a close friend facing the same difficulty." |
| **Mindfulness** | "If I think back to the situation in which I was hard on myself, I was able to tell myself that I was not alone and that other people were going through difficult times just like me" |
