# Supplementary material for: Navigating pathways to develop self-compassion in sport, dance, and music: Qualitative insights from volunteer participants on balancing criticism and compassion, the pivotal influence of the social environment and strategies to overcome setbacks
Source: PLoS One. 2025 Jun 25;20(6):e0326612. doi: 10.1371/journal.pone.0326612 (PMC12194077; doi:10.1371/journal.pone.0326612)
Supplement: S1 Checklist — This is the SwissEthics checklist that regulates research with children and adolescents in Switzerland. (PDF) [file pone.0326612.s003.pdf]

Checklist: Research on and with children and adolescents under the age of 18

The Human Research Act and associated ordinances regulate research with children and adolescents:

Key word	Bases			
	HRA	ClinO	HRO	Explanations
Definition of child/adolescent	Art. 3j, k			
Briefing	Art. 7, 16, 21	Art. 7, 8	Art. 8, 9	Page 19/87
Research with children and adolescents	Art. 22-23			
Emergency situations	Art. 31, no. 3	Art. 15 b		Page 24/87
Further use of data and material	Art. 32-34			Page 60/87
Risk/stress	Art. 22 2a;<br>Art. 23 3a;			
Categorisation (risk)	Art. 45 2	Art. 19-25, 30 ff.	Art. 7, 13	Pages 7, 27-30/87
Register of phase 1 trials				Page 51/87

When assessing research applications involving this age group, it is important to take account of the following:

**1**	**Does the ethics committee possess sufficient paediatric expertise to assess the application?**
**2**	**Are the following conditions satisfied?**
2.1	Can the question only be answered through research on children and/or adolescents?
2.2	Is this a phase 1 trial? (registration obligation)
**3**	**Is the study design appropriate to children?**
3.1	Is it possible to minimise the investigation material (e.g. capillary blood samples)?
3.2	Can the unpleasant aspects be minimised?<br>(e.g. can blood samples be collected only after percutaneous anaesthesia and by experts?)
3.3	What arrangements have been made to avoid pain and anxiety?
3.4	Can the number of test subjects be reduced (statistical analysis)?
3.5	Does the child / adolescent require special monitoring?<br>(e.g. for potential specific, age-related side effects)
**4**	**What is the assessment of the risk-benefit profile?**
4.1	Will the individual benefit directly?<br>• New treatment method with a therapeutic product only available in the study and/or<br>• Participation in the study will optimise comprehensive treatment (e.g. reference appraisal)

4.2	Risk assessment:<br>• Is participation in the study unlikely to exacerbate – and possibly even likely to improve – the child's/adolescent's condition? (If this is not the case, the study is unacceptable)
4.3	Stress assessment:<br>• Are the additional stresses/unpleasant aspects associated with the study tolerable (what reasonable parents would deem to be acceptable for a child of the corresponding age)?
4.4	Is the proposed risk categorisation justified?
4.5	Is the study being conducted under a PIP (Paediatric Investigation Plan) and has it been assessed by an ethics committee?
**5**	**In what form will information be provided?**
5.1	If the trial subjects are **neonates, infants and toddlers,** it will not be possible be brief them. Their legal representatives (usually parents, legal guardians) receive all the information.
5.2	**Children with a developmental age 10 years or under** should be briefed verbally. Their legal representatives (usually parents, legal guardians) receive all the information.
5.3	**Children with a developmental age of 11 - 13** should be given written Patient Information that has been adapted to this age group's comprehension level in addition to a verbal briefing (see Swissethics guidelines)<br>Their legal representatives (usually parents, legal guardians) receive all the information.
5.4	**Adolescents aged 14 and over** receive a verbal briefing and written information with the same content as that given to their legal representative (usually parents, legal guardians), but with a modified form of address. If this is considered appropriate, the polite form of address may be used.
**6**	**Does information need to be provided in stages (e.g. at randomisation)?**<br>• Is this described in the protocol?
**7**	**Might it be appropriate to provide information in another form than text?**
**8**	**Who should sign the consent form?**
8.1	As a rule, the consent of a legal guardian is sufficient (ZGB Art 304, 378 para. 2)
8.2	**Adolescents aged 14 and over** should also sign the consent form.
8.3	A research project in adolescents who are capable of judgement, which entails minimal risks, does not require the informed consent of the legal representatives (parents) (see art. 23 par. 1 lit. b HRA e contrario).
8.4	The legal representatives should provide their consent on the same consent form as that used to confirm that a verbal briefing has been given (swissethics template: Declaration of consent).
